# Response to COVID-19 in Lebanon: update, challenges and lessons learned

**DOI:** 10.1017/S0950268823000067

**Published:** 2023-01-16

**Authors:** Farouk F. Abou Hassan, Mirna Bou Hamdan, Farah Ali, Nada M. Melhem

**Affiliations:** 1 Medical Laboratory Sciences Program, Division of Health Professions, Faculty of Health Sciences, American University of Beirut, Beirut, Lebanon; 2 Department of Epidemiology and Population Health, Faculty of Health Sciences, American University of Beirut, Beirut, Lebanon

**Keywords:** COVID-19, epidemiology, Lebanon, preparedness, response

## Abstract

The COVID-19 pandemic remains a public health problem threatening national and global health security. The socio-economic impact of COVID-19 was more severe on developing countries including Lebanon, especially due to the fragile healthcare system, weak surveillance infrastructure and lack of comprehensive emergency preparedness and response plans. Lebanon has been struggling with plethora of challenges at the social, economic, financial, political and healthcare levels prior to the COVID-19 pandemic. The COVID-19 pandemic in Lebanon revealed gaps and challenges across the spectrum of preparedness and response to emergencies. Despite these challenges, the Lebanese response was successful in delaying the steep surge of COVID-19 cases and hospitalisations through imposing strict public health and social measures. The deployment of the national vaccination plan in Lebanon in February 2021 coincided with the reduction in the number of cases and hospitalisation rates. The aim of this manuscript is to advance the epidemiologic evolution of COVID-19 in Lebanon pre- and post-vaccination, the challenges affecting the response and recovery, and the lessons learned.

## Introduction

Since its emergence in December 2019, severe acute respiratory syndrome coronavirus 2 (SARS-CoV-2), the causative agent of coronavirus disease 2019 (COVID-19), has been a global public health problem. SARS-CoV-2 has rapidly spread worldwide and accounted for more than 649 711 690 cases and 6 654 580 deaths until the write-up of this manuscript [1]. In Lebanon, more than 1 221 225 confirmed cases and 10 740 deaths have been reported [2].

Lebanon is a small country (10 452 km^2^) in the Eastern Mediterranean Region (EMR) with an estimated population of 6.7 million [3]. The country hosts the largest number of refugees per capita and per square kilometre in the world with an estimate of 1.5 million Syrian refugees, 210 000 Palestinian refugees and more than 13 700 refugees of other nationalities [4, 5]. This increased the burden of the national economy and weakened the already fragile healthcare system. October 2019 marked the beginning of a severe and persistent economic and financial crisis in Lebanon [6], which was exacerbated by the COVID-19 pandemic. The World Bank described this economic and financial crisis among the worst globally since the mid-19th century with an estimated nominal GDP of $23.1 billion in 2021 compared to $52 billion in 2019; with the Lebanese pounds losing more than 90% of its value against the US dollar [7]. During the last couple of years, the banking sector implemented informal capital controls due to the depletion of foreign exchange (FX) reserves; consequently, Lebanon witnessed a drastic collapse in basic services and inability to import basic goods including necessary medical devices and essential medications amidst a global pandemic; consequently, the Lebanese government was short on funds and lacked the ability to equip public and private hospitals with the needed supplies [8, 9]. This resulted in the complete dependence of some hospitals on the World Health Organization (WHO) and on foreign and local non-governmental aids to equip their premises with essential medical supplies and equipment [10].

Early during the pandemic and prior to the development and deployment of vaccines, the Lebanese government declared a state of emergency and imposed strict public health and social measures (PHSMs) to reduce the spread of SARS-CoV-2. These included, similar to other countries, points-of-entry closure, travel restrictions and complete or partial national lockdowns, in addition to face masks mandate, social distancing and quarantining following exposure to a suspected or confirmed COVID-19 case [11, 12]. These non-pharmaceutical interventions (NPIs) aimed at mitigating and delaying the steep rise in the number of cases and hospitalisation rates, thus reducing the pressure on healthcare systems and healthcare workers (HCWs). However, these measures were coupled with economic and social implications including high rates of unemployment and closure of small businesses [10, 13, 14]. Global economy and financial markets were severely affected by these mitigating strategies and resulted in significant reductions in income, increase in unemployment rates, severe disruptions in transportation and manufacturing industries and immense disruption in trade and investment [15, 16]. The socio-economic impact of COVID-19 was more severe on developing countries including Lebanon, especially due to the fragile healthcare system, weak surveillance infrastructure and lack of comprehensive emergency preparedness and response plans. Although the global economy started to recover from COVID-19with 5.6% growth in the world economy in 2021, developing countries including Lebanon were much less able to recover [16].

The purpose of this manuscript is to advance the evolution of the COVID-19 epidemiology in Lebanon as well as gaps and challenges in the management of and response to the pandemic.

## National response to COVID-19 prior to vaccination

### Impact of implementation and relaxation of public health and social measures

**Fig. 1.**
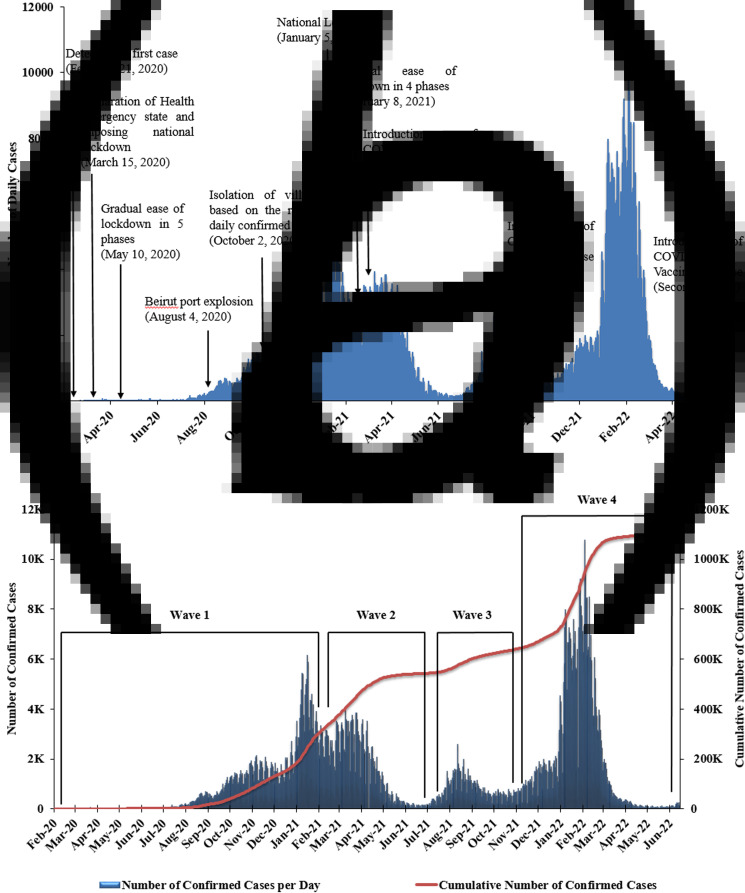
COVID-19 in Lebanon. (a) The national response to the COVID-19 pandemic in Lebanon (February 2020–June 2022) included different public health and social measures implemented before and after the introduction of COVID-19 vaccines impacting the number of daily confirmed SARS-CoV-2 cases ; *X*-axis: month-year; *Y*-axis: daily number of confirmed cases. (b) The daily and cumulative number of COVID-19 cases throughout the four waves of the pandemic in Lebanon (February 2020–June 2022); *X*-axis: month-year; *Y*-axis left: number of confirmed daily cases; *Y*-axis right: cumulative number of confirmed cases during the same period.

Since the detection of SARS-CoV-2 in Lebanon, the Lebanese government dealt with the pandemic through intermittent lockdowns and other mitigation strategies to reduce community transmission. These included, similar to other countries, the implementation of travel bans, declaration of a state of national emergency with a complete national lockdown imposed by mid-March 2020, only few days following the declaration of the pandemic by the WHO [17, 18]. In order to limit general mobilisation during the lockdown while maintaining the sustainability of essential services, the Lebanese government successfully enforced traffic regulations based on odd/even rationing of vehicles (5 April 2020–14 June 2020) [19]. The government was successful at the implementation of NPIs (national lockdowns, social distancing and case isolation) as well as mask mandates; the latter were imposed on 29 May 2020 [20]. These measures delayed the dramatic surge in the number of cases and deaths; importantly, these measures were instrumental to prepare healthcare centres to isolate and treat COVID-19 cases, to scale-up surveillance and contact tracing as well as testing and diagnostics capacity (Fig. 1a). This was clearly reflected through the low number of cases reported until the end of May 2020 (Figs 1a and 1b).

**Fig. 2.**
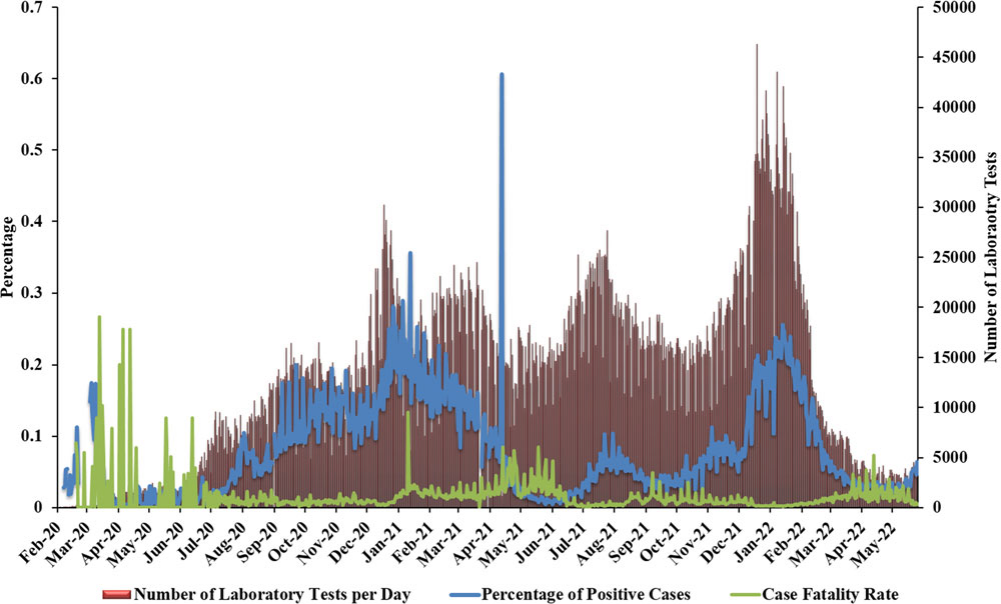
COVID-19 positivity rates and case fatality rates in Lebanon (February 2020–June 2022). This figure displays the scale-up of PCR testing in Lebanon, and the evolution of positivity and case fatality rates across time. *X*-axis: month-year; *Y*-axis left: percentage (PCR positivity rate and CFR); *Y*-axis right: number of daily PCR tests.

At the beginning of the pandemic, the Rafik Hariri University Hospital (RHUH), a public hospital, was the only hospital admitting and caring for COVID-19 patients. However, the first national lockdown (March–May 2020) allowed other major academic hospitals to establish COVID-19 units. During this time, the Ministry of Public Health (MoPH) equipped other public hospitals and healthcare facilities across Lebanon with personal protective equipment (PPEs) and ventilators to provide care for hospitalised patients [21]. The national lockdown implemented between March and May 2020 also resulted in the preparedness of 47 laboratories nationwide for COVID-19 testing, 1365 hospital beds for patients and a ratio of 21 beds per 100 000 for critical care in addition to the increase in the number of ventilators to a total of 1424 (20% increase) [21]. Between April and May 2020, the average positivity rate was 1.2% (Fig. 2). This low positivity rate could be attributed to the lockdown, the low number of laboratory tests performed in addition to the lack of national mass testing programmes amid a national economic crisis. Towards the end of April 2020, a five-phase plan for the ease of the national lockdown was put in place. This strategy aimed to gradually lift the lockdown while monitoring its impact on the levels of community transmission. The first phase started on 27 April 2020 and included the opening of vital economic sectors with low risk of viral transmission. Ten to 14 days later, the lockdown was eased whereby sectors with higher risk of transmission were reopened in the second and third phases [21]. The fourth phase targeted the reopening of the retail and recreational centres on 8 June 2020 and the final phase targeted the lift of international travel bans on 1 July 2020 [22]. The gradual lift of the lockdown resulted in a 2.5-fold increase in the number of confirmed COVID-19 cases, 13.5-fold increase in the positivity rate and onefold increase in case fatality rates (CFR) by the end of July 2020 compared to the end of April 2020.

### Impact of political and socio-economic dynamics on the epidemiology of SARS-CoV-2 and the national response to the pandemic

The explosion of the Beirut port on 4 August 2020 resulted in more than 220 deaths, 6500 wounded and left 300 000 people displaced [23]. Importantly, this blast impacted half of the capital's healthcare centres amidst a growing pandemic leading to weakening of an already fragile healthcare system [24]. Two weeks following the blast, a three- and fourfold increase in the number of daily reported cases and testing positivity rates were reported, respectively (Figs 1b and 2). Moreover, the cumulative number of COVID-19 deaths per month increased to 108 in August 2020 compared to a total of 25 deaths in July 2020. This is especially due to the shifted national attention from COVID-19 to caring for the blast victims. In an attempt to reduce the impact of the above on the fragile healthcare system, a 2-week lockdown was enforced (5 days of full lockdown followed by 2 days of partial lockdown repeatedly) between 21 August and 3 September 2020 [25]; however, these measures did not reduce the community spread of COVID-19 which kept on the rise with an average daily cases of 744 and 1362 in September and October 2020, respectively. The positivity rate also followed similar trends with a daily average of 7.2% in September and 11.4% in October 2020.

**Fig. 3.**
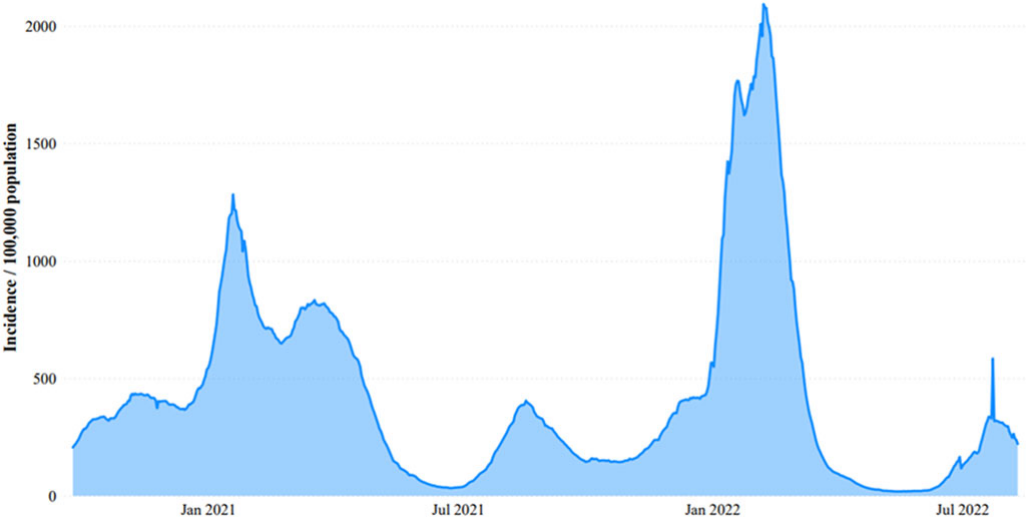
COVID-19 incidence rates in Lebanon (January 2021–July 2022). This figure displays the changes in COVID-19 incidence rates per 100 000 population across time. *X*-axis: month-year; *Y*-axis: incidence per 100 000 population.

The continuous surge of COVID-19 cases and lack of compliance with PHSMs were the main drivers to introduce a new approach to manage and further reduce community spread. In October 2020, the government started isolating villages based on the average rate of daily cases per 100 000 residents over 14 days (Fig. 1a) [26]. Based on the latter, the risk level of transmission per district was divided into three different levels: low (<4 cases), moderate (4–8 cases) and high (>8 cases) [27]. Consequently, a complete lockdown of more than 160 villages was implemented by mid-October; the former was accompanied by the closure of pubs, bars and night clubs and the enforcement of mandatory indoors face mask wear nationwide [28]. This strategy was supported by the engagement of a large number of municipalities specifically in implementing and monitoring isolation of positive cases as well as reporting. This ‘green zoning’ approach has been previously implemented in many European countries during the COVID-19 pandemic in order to ease the economic impact of blanket lockdowns as well as the spread of the virus by implementing mobility restrictions based on the epidemiologic status of these zones [29]. In Lebanon, the implementation of this approach did not dramatically change the CFR nor effectively slowed the level of community transmission whereby the 14-day average incidence rate increased from 206 to 433 per 100 000 population between 25 September 2020 and 14 November 2020, respectively (Fig. 3). The ‘green zoning’ approach led to the interruption of businesses which subsequently led to protesting the negative impact of these measures in the absence of incentives and subsidies, especially to small businesses. The zoning approach exacerbated inequalities among different regions in the middle of an economic crisis. Consequently, the government imposed a 2-week national lockdown on 14 November 2020, prior to the holiday season (Fig. 1a). During that time, the level of community transmission (CT) reached its highest level (CT4). The CT level was based on the WHO criteria whereby community transmission level 1 (CT1), CT2, CT3 and CT4 are defined as low, moderate, high and very high incidence of locally acquired widely dispersed cases detected in the past 14 days, respectively. These levels took into account the incidence rate, deaths, hospitalisations as well as testing positivity rates [30].

**Fig. 4.**
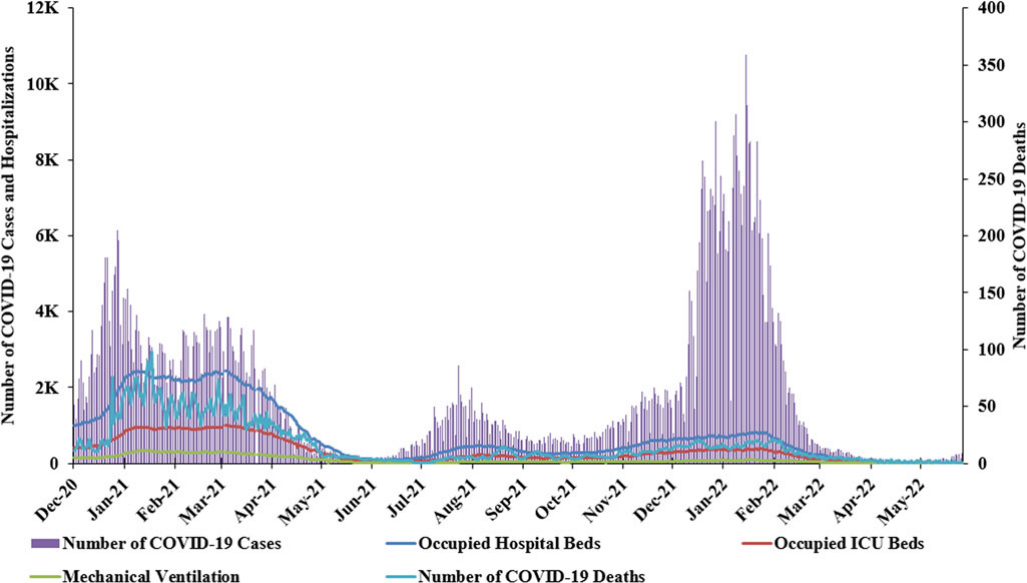
COVID-19-assocaited hospitalisation in Lebanon (December 2021–June 2022). This figure shows the hospitalisation caused by COVID-19 before and after the introduction of vaccines while featuring the number of occupied hospital beds (blue line), occupied ICU beds (red line) and patients under mechanical ventilation (green line). *X*-axis: month-year; *Y*-axis left: number of confirmed daily cases, ICU patients, hospitalised patients and patients on mechanical ventilation; *Y*-axis right: number of daily COVID-19 deaths.

The lifting of PHSMs prior to the availability of COVID-19 vaccines, and specifically during the 2020 holiday season, had a significant impact on the already fragile healthcare system suffering from shortage of medical equipment and medication as well as incentives and livelihood subsidies. This coincided with the detection of the first SARS-CoV-2 variant of concern (VOC), alpha (B.1.1.7) [31]. A sharp and unprecedented rise in the number of COVID-19 cases, hospitalisations and deaths in Lebanon was observed with an average daily reported case surpassing 3800 (Fig. 1b), an average daily positivity rate of 20% (Fig. 2) and an incidence rate of 867/100 000 population (Fig. 3). Moreover, the average number of daily deaths almost quadrupled compared to the period extending between September and December 2020. Concurrently, this peak was coupled with 1868 occupied hospital beds, 715 occupied intensive care unit (ICU) beds and 226 patients on ventilators per day; this translated into ICU beds occupancy of more than 90% nationwide (Fig. 4) [31]. The sharp increase in morbidity and mortality prompted the government to impose a strict nationwide lockdown on 15 January 2021, which extended until 8 February 2021 (Fig. 1).

## National response and COVID-19 vaccines

### Vaccination plan in Lebanon

The day 27 January 2021 marked the announcement of the national COVID-19 vaccination plan as well as the launching of an online registration platform, IMPACT, for COVID-19 vaccine registration and reporting of adverse events [32]. The COVID-19 Vaccine National Coordinating Committee focused on the main pillars for Vaccine Introduction Readiness Assessment Tool/Vaccine Readiness Assessment Framework (VIRAT/VRAF): prioritisation, targeting and population calculation, service delivery, vaccine supply chain and logistics, regulatory pathways, training, supervision and communication, resources and funding, safety surveillance, monitoring and evaluation [33, 34]. The plan aimed to vaccinate 70% of the population by the end of 2022, reduce the spread of the virus and decrease morbidity and mortality rates and the need for critical care among priority groups with high risk of complications due to SARS-CoV-2 infection. The vaccination priority groups were originally categorised based on risk of exposure and infection, risk of complications following infection, essential personnel (front-line responders to the pandemic, workers in primary healthcare centres) and the availability of vaccines (Table 1) [32, 35]. The start of the vaccination campaign on 14 February 2021 coincided with the gradual ease of the national lockdown mentioned above and the global circulation of the Beta VOC (B.1.351) (Fig. 1a).

**Table 1.** Initial phases and target groups of the national COVID-19 vaccination plan

Phases	Targeted groups
Phase I	
Phase IA	Healthcare workers All individuals ≥75 years old
Phase IB	All individuals 65–74 years old All individuals 55–64 years with comorbidities Individuals working in epidemiological surveillance and MoPH
Phase II	
Phase IIA	People 55–64 years that have not yet been vaccinated in phase IB Individuals 16–54 years with comorbidities and workers in primary healthcare centres that haven't been vaccinated in Phase I
Phase IIB	Essential personnel in the public sector and those working in elderly care homes People with special needs inmates
Phase III	Teachers and staff of kindergartens and schools Workers in areas with high risk of infection and people who care for members of their family that are ≥65 years or with special needs
Phase IV	All individuals willing to receive the vaccine

The BNT162b2 vaccine was the first to be delivered to Lebanon followed by the ChAdOx1 vaccine [36, 37]. By the end of April 2021, the rate of first dose vaccine coverage remained low at 6.3% [38]. In an effort to increase vaccine coverage and speed up vaccination rollout, the MoPH mobilised the private sector in the procurement of vaccines, and signed an agreement with Pfizer-BioNtech to receive vaccine doses aiming at vaccinating students, faculty and staff for a safe return to campuses starting fall 2021 [39–41]. In addition, a large awareness campaign, vaccine administration on a walk-in basis and organising vaccine marathons were implemented [42, 43]. These measures increased the first dose coverage by approximately fourfold [2]. Moreover, by the end of October 2021, vaccination campaigns targeting students ≥11 years old in public and private schools were launched [44]. In December 2021, more than 613 000 doses of Johnson & Johnson (J&J) and Moderna vaccines were donated to Lebanon through the COVAX platform [45]. As of 12 December 2022, the vaccine coverage for the first dose was 50.3% and that of the second dose was 44.3%. Of those who received the second dose, only 27.3% received the third dose of the vaccine [46]. Unfortunately, and despite all efforts to increase vaccine uptake, the country is still far from the 70% target of vaccine uptake by the end of 2022 as per the vaccination plan.

### Impact of vaccination and emergence of new SARS-CoV-2 variants on community transmission

Despite all the challenges, the national vaccine campaign was successfully launched and was coupled with a gradual decline in the number of reported cases and incidence rates. The lowest number of reported cases was reported in the period extending between 1 May and 31 July 2021 with an average daily case of 378 and average daily positivity rate of 2.3% (Figs 1b and 2). Similarly, the average daily number of patients in regular hospital beds, ICU beds and on mechanical ventilation were 321, 160 and 50, respectively (Fig. 4). This is equivalent to 83% reduction in the average number of patients requiring hospitalisation per day and 78% reduction in patients requiring ICU admission and mechanical ventilation compared to January 2021. The decline in COVID-19 cases during that time could be attributed to multiple factors in addition to COVID-19 vaccines. These include the continuous remote schooling and working from home and the increased use of masks and social distancing practices in addition to natural immunity acquired following infection.

With the emergence of SARS-CoV-2 variants and vaccine escape mutants, several studies reported breakthrough infections among fully vaccinated individuals [47–52]. However, the majority of breakthrough infections among vaccinated individuals tend to be mild or asymptomatic [50]. Importantly, unvaccinated individuals were 3.5 and 21 times at higher risk of testing positive for SARS-CoV-2 or die from COVID-19, respectively, compared to individuals vaccinated with two doses and boosted [53]. In Lebanon, and following vaccination, we observed a dramatic decrease in hospitalisation rates during the third and fourth waves of the pandemic compared to the second wave prior to vaccination (Figs 1b and 4). During the third wave (July–October 2021) when the Delta VOC was predominantly circulating worldwide, the average number of daily cases reached a peak in August and September with 1321 and 2085, respectively (Fig. 1b). A similar pattern was observed in relation to the average daily positivity and incidence rates (Figs 2 and 3). During the fourth wave (December 2021–January 2022) when the Omicron VOC took over, there was a sharp increase in the above parameters (Fig. 1b). Similar to the first wave, this sharp increase in the number of cases in January 2022 was driven by the holiday season and the mass gatherings that took place during New Year's Eve. This is also attributed to the emergence of new and highly transmissible Omicron sub-lineages namely BA.1 and BA.2 which accounted for 78% and 16% of sequenced samples submitted to GISAID between December 2021 and January 2022 [54]. This is in accordance with recent data from our group that showed that among 250 sequenced samples from HCWs in Lebanon between December 2021 and January 2022, 57% of them tested positive for Omicron sub-lineage BA.1.1 and 19% Omicron sub-lineage BA.1 [55]. During that time, both the average number of daily cases and positivity rates increased from 1848 and 8.4% in December 2021 to 6235 and 18.4% in January 2022, respectively (Figs 1b and 2). The average 14-day incidence rate also jumped from 411/100 000 in December 2021 to 1381/100 000 in January 2022 (Fig. 3). Despite these increases, we observed a decline in hospitalisation rates (Fig. 4), especially due to the increased vaccine coverage during that time (47% first dose and 39% second dose) [2]. During the fourth wave, 81% of patients in ICU or requiring mechanical ventilation were unvaccinated underscoring the ability of vaccines to prevent severe illness and highlighting the importance of scaling up vaccination coverage across all age groups.

## National challenges and successes

### Epidemiologic and genomic surveillance of SARS-CoV-2

At the beginning of the pandemic in Lebanon, there was limited capacity in detecting, tracing and isolating confirmed COVID-19 cases. The RHUH was the only hospital designated for testing, quarantining and treating confirmed cases [21]. This resulted in an underestimation of community spread and the inability to predict trends and thus to properly inform decision-making. This limited capacity was mainly due to an under-resourced and under-staffed surveillance system, delayed scale-up of diagnostic capacity, lack of isolation facilities, lack of data sharing and established decentralised health informatics, and lack of digital contact tracing through mobile applications; the latter was used in many countries globally such as the USA [55] and other countries in Asia [57, 58], Europe [59], and the region specifically Jordan [60], United Arab Emirates (UAE) [61], Qatar [62], Oman [63], Kuwait, Algeria, Tunisia, Bahrain [64] and Saudi Arabia [65]. Moreover, the heavy socio-economic impact of national lockdowns affected underprivileged individuals, especially due to the economic crisis the country is suffering from.

SARS-CoV-2 seroprevalence studies are important to estimate the burden of COVID-19 in a population and to understand the dynamics of disease transmission, risk of infection as well as the evolution of the pandemic [66]. While serosurveys are important to guide the public health response, unfortunately, few seroprevalence studies on anti-SARS-CoV-2 antibodies were performed in Lebanon with most of them using sera collected early during the pandemic [67–69]. The results of these studies showed a gradual increase in seropositivity from 2.7% (November 2020) to 84.8% (April 2021) [67, 69]. This increase was expected due to the unprecedented rise in the number of COVID-19 cases detected during the same period (Fig. 1a). Importantly, variable seropositivity rates were reported in different districts and towns in Lebanon [69]; with data revealing an insufficient seroprevalence to promote protection following natural infection. Importantly, longitudinal studies are needed especially following the administration of COVID-19 vaccination.

Moreover, the lack of concerted efforts between academics and national stakeholders as well as lack of information-sharing system resulted in poor engagement of the scientific community in supporting decision-making. Modelling studies, for examples, are important to inform and help policy makers to implement appropriate public health measures and mitigate virus spread. Few modelling studies were conducted in Lebanon to predict the spread of SARS-CoV-2 as well as the impact of COVID-19 vaccination [40, 70, 71]. An early modelling study simulated a sharp increase in the number of cases and deaths if schools and universities were open when vaccination rates did not exceed 4% in April 2021 [70]. While these studies are instrumental to guide public health policies during public health emergencies, they are hampered in countries like Lebanon by the lack of data and information-sharing systems and thus the utility of these studies. More studies are needed in collaboration with the MoPH in order to predict disease trends as well as hospitalisation for better preparedness to pandemic threats.

Importantly, limited genomic data on circulating SARS-CoV-2 variants have been available in Lebanon due to the lack of national genomic surveillance programmes and the lack of concerted efforts between research institutions and the MoPH to support the latter. SARS-CoV-2 genomic surveillance is important to continuously monitor the circulation and emergence of variants in order to guide national public health policies. Scattered reports on limited number of samples revealed the detection of B.1 lineage (20A clade), B.4 lineage (19A clade) and the B.1.1 lineage (20B clade) in 11 samples collected from hospitalised patients between February and March 2020 [72]. Later and until January 2021, B.1.398 followed by B.1.1.7 and B.1 SARS-CoV-2 lineages was detected and reported among 58 patients [73]. Acknowledging the urgent need for SARS-CoV-2 national genomic surveillance programmes to inform public health strategies, we established a national SARS-CoV-2 genomic surveillance to continuously monitor circulating and emerging variants among HCWs and hospitalised patients in Lebanon in collaboration with the MoPH and its epidemiologic surveillance unit. Our data were similar to global trends whereby Omicron sub-lineage BA.1.1 followed by BA.1 were predominantly circulating between December 2021 and January 2022 [55], followed by a shift in the predominance to BA.2 between February and March 2022; the latter was substituted by BA.5 sub-variant between March and September 2022 (*unpublished data*).

### The healthcare system

The structure of the national healthcare system had a major impact on the national response to COVID-19. Approximately, 84% of healthcare facilities are located in large cities and most of them are private healthcare facilities [21]. The number of public hospitals is limited and constitutes 15% of total number of hospital beds; these hospitals are mostly not well equipped, understaffed and underfunded compared to private hospitals [74]. In April 2020, experts supporting the government and the MoPH developed a hospital emergency preparedness plan to assess the gaps and analyse needs of public hospitals in order to prepare these hospitals and increase their capacities during the pandemic and prepare them for future ones. The assessment identified the need for diagnostics, PPEs, extra beds and ventilators in addition to human resources. Early during the pandemic, the limited engagement of the private healthcare sector coupled with the lack of public–private partnership, low healthcare budget offered to public hospitals, as well as lack of COVID-19 units and ICU beds in private hospitals hampered early responses. These factors along with the financial crisis exerted a heavy burden on hospital staff which clearly led to a brain drain of health workforce at an alarming rate of 40% and 30% of skilled medical doctors and registered nurses, respectively, estimated to have left the country permanently or temporarily by September 2021 [75].

Despite all these challenges, Lebanon was able to delay the exponential rise in COVID-19 cases until January 2021 through intermittent lockdowns and community mobilisation. The latter was translated by the engagement of municipalities in sharing information with the MoPH, albeit later during the course of the pandemic, as well as monitoring home isolation for confirmed and suspected cases under their jurisdiction. Few months into the pandemic, the concerted efforts and collaboration between the MoPH, the Ministry of Interior, the Lebanese Red Cross, the United Nations agencies, the WHO and the Disaster Risk Management Unit resulted in better implementation of the national response against the spread of SARS-CoV-2. Moreover, volunteers were trained for contact tracing nationwide, testing and data collection specifically at points of entry (airport and land borders). The ability of the RHUH to care for COVID-19 patients early during the pandemic, the compliance of the Lebanese community to the implemented national strategies and safety measures, and the deployment of the COVID-19 vaccines contributed significantly to the success of the national response.

## Lessons learned and recommendations

The COVID-19 pandemic revealed global gaps and brought to the surface the need to reimagine the pillars of global health security. Lebanon, similar to other countries, grappled with a variety of challenges that unravelled the dire need for a national strategy cutting across emergency preparedness, response and recovery from crises. This strategy should be applied and sustainable for any type of threat including agents of potential epidemic threats as well as biological, chemical, radiologic and natural disasters. A prioritised plan of action supporting the development of the national health sector strategy on health security as well as strengthening the existing infrastructure is needed. These efforts require rethinking and reimagining the healthcare system and its components in order to manage crises. The COVID-19 pandemic unravelled many structural obstacles and barriers spanning the entire spectrum of emergency preparedness in Lebanon. The need to enhance surveillance and shift from disease-based surveillance into comprehensive surveillance programmes was clear for timely response to disease outbreaks. While this might seem generic, however, Lebanon has been suffering from political instability for the past five decades; the former hampered the institutionalisation of systemic programmes for disease prevention and care. Consequently, political commitment is needed to invest in sustainable capacity building and strengthening and updating the infrastructure of Lebanon's surveillance system and its healthcare infrastructure through: establishing an organic and sustainable link between the public and private sectors, the development of laboratory networks and decentralised yet linked genomic surveillance programme, robust health bioinformatic system for transparent data sharing while linking epidemiologic data to clinical data and genomics; importantly, the coordination between policy makers and research institutions is critical to advance contextual data that will feed into national needs of national stock supplies and supply chain (medicines, vaccines, diagnostics, PPE and others). While researchers are well represented on national, regional and international committees and task forces on prevention and control of infectious diseases and disaster management and while the former had a positive impact on guiding national policies to control COVID-19 in Lebanon, an inherent lack of trust between policy makers and the community was obvious through lack of compliance of the community and lack of catch-up on vaccination depicted through the current low coverage rates. The development of a strong risk communication and community strategy is instrumental in times of emergencies and allows the engagement of communities in policymaking. This is more critical in Lebanon in order to build trust and engage the public in decision-making.

Importantly, the recovery from an emergency must begin during the response and consequently, we recommend the development of a national policy for safe return to work, the establishment of a multisectoral recovery task force to coordinate and implement recovery arrangements (societal, financial, health-related, among others), and supporting the MoPH to expand resources to strengthen the medical and public health sectors.

## Conclusion

Enormous efforts and actions were put in place to respond to the COVID-19 pandemic in Lebanon during a progressively worsening economic and financial situation; however, the COVID-19 pandemic revealed the vulnerability, gaps and needs of the healthcare system in Lebanon including epidemiologic surveillance, genomic surveillance, integrated and concerted data sharing, diagnostic capacity, community mobilisation and risk communication. The national commitment to these important inherent components of a rapid response requires resources and investment in human and technical expertise to reduce inequality in access to information and care.

## Data Availability

The authors confirm that the data supporting the findings of this study are available within the article.
